# Are biological agents toxic to human chondrocytes and osteocytes?

**DOI:** 10.1186/s13018-015-0264-y

**Published:** 2015-07-30

**Authors:** Mehmet Isyar, Bulent Bilir, Ibrahim Yilmaz, Selami Cakmak, Duygu Yasar Sirin, Aliye Yildirim Guzelant, Mahir Mahirogullari

**Affiliations:** Department of Orthopaedic and Traumatology, School of Medicine, Istanbul Medipol University, Bagcilar, 34214 Istanbul, Turkey; Department of Internal Medicine, School of Medicine, Namik Kemal University, 59100 Tekirdag, Turkey; Department of Pharmacovigilance and Rational Drug Use Team, Republic of Turkey, Ministry of Health, State Hospital, 59100 Tekirdag, Turkey; Department of Orthopaedic and Traumatology, Haydarpasa Training Hospital, Gulhane Military Medical Academy, 34668, Istanbul, Turkey; Faculty of Science, Department of Molecular Biology and Genetics, Namik Kemal University, 59100 Tekirdag, Turkey; Department of Physical Medicine and Rehabilitation, School of Medicine, Namik Kemal University, 59100 Tekirdag, Turkey

**Keywords:** Biological agents, Chondrotoxicity, Osteotoxicity, Primary cell culture

## Abstract

**Purpose:**

The aim of the present study is to investigate the effects of biological agents (BAs) on human chondrocytes and osteocytes *in vitro.*

**Methods:**

Primary cell cultures obtained from gonarthrosis patients were divided into four groups, two of which were designated as control cultures of chondrocyte and osteocyte, and the other two groups were exposed to BAs administered via the culture medium. Cultured cells were characterized by immunophenotyping. Before and after administration of the agents, the cultures were observed by inverted and environmental scanning electron microscopy (ESEM). The number of live cells and the proliferation rate were monitored by MTT assay.

**Results:**

Rituximab and adalimumab were the least toxic agents to chondrocytes, whereas adalimumab and etanercept were to osteocytes.

**Conclusion:**

During periods of intense active inflammation, the concentration of the preferred BAs after inhibition of inflammation needs to be emphasized when their effects on cartilage and bone tissue are considered at the cellular level if the clinical practice is to continue.

## Introduction

Rheumatic diseases are systemic inflammatory conditions that may cause severe joint degeneration and are associated with high morbidity and mortality rates [1]. In recent years, in an attempt to prevent joint degeneration associated with rheumatic diseases, drugs known as biological agents (BAs) have been approved for clinical use in the treatment of rheumatic diseases, and their usage is increasing [2]. Recently, personalized medical treatment has become more desirable, making the careful identification of the adverse effects of BAs a matter of great importance [1].

Disease-modifying anti-rheumatic drugs are declared as the traditional treatment of rheumatic diseases and, even if these drugs reduce the symptoms of the disease in a long-term, they have limited effects to terminate the disease completely [3]. Involved in exceeding this limit, BAs, which are products of the developing biotechnology, have become the hope for the prevention of inflammatory diseases [4], and the use of high-quality tumor necrosis factor (TNF)-α inhibitors, interleukin (IL-1) blockers, anti-B cell (CD20) antibody, and T cell co-stimulatory modulators has created a revolution in the treatment [5]. However, although the efficiency of these agents in the treatment is well known, they also cause serious side effects because of their immunosuppressive activity [6–11], and hence, the necessity of a comparison of side-effect profiles based on clinical observations is emphasized [7]. Indeed, planning a treatment at the cellular level to regain the functionality of tissues or organs is mentioned nowadays, as indicated in literature, and a comparison of clinical observations may not be sufficient. Therefore, additional researches based on cellular level and pharmaco-molecular approach are still needed, before clinical applications of BAs.

In the present study, we aim to observe the effects of three TNF inhibitors, etanercept (ETA) (soluble TNF receptor fusion protein (p75-IgG fusion protein)), infliximab (INF) (chimeric anti-TNF monoclonal antibody), and adalimumab (ADA) (recombinant human IgG1 monoclonal antibody), and two IL-1 antagonists, abatacept (ABA) (CTLA4-IgG1 fusion protein) and rituximab (RIT) (anti-CD20 monoclonal antibody; used in B-cell-inhibiting treatments on human primary chondrocytes and osteocytes *in vitro*). Using these methods, we aim to evaluate the toxicity and effects of the BAs on the viability and proliferation of the cultured cells.

## Methods

### Ethics approval and permission

This scientific research project (2014-28/23) was approved by the Local Scientific Research Ethics Committee of Gulhane Military Medical Academy (GMMA) on February 27, 2014, with the authorization number 1491–29–14/1539. To perform molecular analyses on cellular material, written informed consent was obtained from patients. Specific BAs were numbered and researchers were blinded to each specific BA added to each culture medium. All experiments were carried out at least three times.

### Patients and settings

**Fig. 1:**
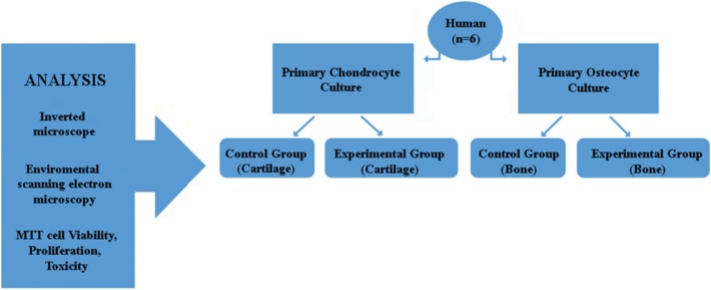
Experimental study design

Patients with a history of methotrexate, fludarabine, cyclophosphamide, and high-dose steroid use, and those who received an anti-pneumococcal vaccination in the preceding 3 years were excluded from the study. Tissues obtained from the patients with confirmed gonarthrosis (*n* = 6, mean age 65 years; stage 4 gonarthrosis according to the Kellgren-Lawrence protocol [12]) were included in the study, and the lack of protein allergy in their anamnesis was confirmed. Tissues resected from the distal femur were divided into two groups to establish primary chondrocyte and osteocyte culture. The experimental study design and workflow are summarized in Fig. 1.

### Materials

Collagenase type II enzyme (1 g, catalog # 17101–015, Gibco), Hank’s balanced salt solution (HBSS-1X, catalog # 14025, Gibco), penicillin-streptomycin (PS), fetal bovine serum (FBS), and Dulbecco’s modified Eagle’s medium (DMEM 1000 mg glucose/L) were obtained from Sigma Chemical, St. Louis, USA. Insulin-transferrin-selenious acid (ITS) premix was obtained from Sigma-Aldrich GmbH, Germany. MesenCult Osteogenic Stimulatory Kit for MesenCult-XF (catalog # 05434, containing MesenCult Basal Medium, catalog # 05431, Osteogenic Stimulatory Supplement, catalog # 05435, and β-Glycerophosphate, catalog # 05436) were obtained from STEMCELL Technologies, Canada. MTT assays were performed using the Vybrant MTT Cell Proliferation assay (catalog # V-13154), obtained from Cell Biolabs Inc., USA. The immunoflow cytometer Beckman Coulter (catalog # Navios) was obtained from France. Immunoflow CD cell surface markers were performed using the Beckman Coulter Immunotech company, obtained from France (catalog # A07765). The laminar flow cabinet (Air Flow-NUVE /NF–800 R) and incubator (NUVE, 06750) were obtained from Ankara, Turkey. An Olympus CKX41 inverted microscope was used for light microscopy, in conjunction with the Olympus Cell Soft Imaging System software. A Mindray MR-96A (China) ELISA was used for viability and cytotoxicity measurements, while a Quanta 250 FEG (Fei Company, Hillsboro, OR, USA) environmental scanning electron microscope (ESEM) was used for electron microscopy.

### Preparation of BAs

RIT, ADA, ABA, ETA, and INF were stored in a laminar flow cabinet and dissolved using appropriate solvents (0.9 % isotonic or lactated ringer or its solvents) to obtain stock solutions at concentrations of 250, 500, 40, 50, and 100 mg/mL, respectively. Solutions were transferred into opaque bottles, prior to labeling to be transferred to the researchers. To apply those BAs to the primary cell cultures, each stock solution was finally diluted with culture medium to reach a final concentration of 10 μg/mL, which is consistent with the standard clinical dosage. Concentrations of commercial stock solutions, applied volumes, and final concentrations are given in Table 1.

**Table 1:** Biological agents, commercial stock solution concentrations, applied volumes, and final concentrations

BA	Commercial stock solution concentration (mg/mL)	Applied volumes (μL/mL)	Final concentration (μL/mL)
Rituximab (RIT)	250	1	10
Adalimumab (ADA)	500	0.2	10
Abatacept (ABA)	40	1	10
Etanercept (ETA)	50	0.4	10
Infliximab (INF)	100	0.8	10

One well 3 mL in volume will allow the BA application

### Primary cell culture

Resected tissues from both groups: bone and cartilage were immediately transferred to the laboratory in transfer medium (DMEM containing 1 % PS) under proper conditions (4 °C). In a laminar flow cabinet, samples were irrigated with 0.9 % isotonic sodium chloride solution to remove red blood cells and cut into small pieces approximately 0.25 cm^3^ using a rongeur. Samples were then washed in a 50-mL 0.1 % (a/h) HBSS solution. Cartilage and bone pieces were transferred into separate 50-mL Falcon tubes, labeled, and processed using standard human primary cell culture protocols. To perform enzymatic digestion, 200 units/mL collagenase type II enzyme mixture, dissolved in complete medium, was used. Tissue samples were incubated for 16 h in a CO_2_ incubator. Afterwards, tissue samples were centrifuged at 4 °C at 1200 rpm for 10 min to discard collagenase. Sedimented cartilage and bone tissue pellets were resuspended in fresh culture medium (DMEM), transferred to flasks to obtain primary cultures.

The complete culture medium (FBS, PS, and DMEM) was exchanged every 2 days [13]. Chondrocytes were then maintained in ITS-supplemented DMEM for an additional 14 days [13, 14]. Osteocytes were maintained for 20 days, refreshing the medium (47.5-mL MSC basal medium, 2.5-mL osteogenic stimulator supplement, 175-μL 1 M β-glycerophosphate, FBS, PS, and DMEM) every 2 days [15]. At the end of the incubation period, cells were detached from flasks with trypsin and counted using a Thoma cell counting chamber in the presence of trypan blue. To six well plates, 1.6 × 10^5^ osteocytes and 2.9 × 10^5^ chondrocytes were passaged and returned to the incubator for 24 h, prior to use in exposure experiments.

### Preparation of cultures for ESEM

The culture medium was removed from the plates using a gun pipettor. A 2.5 % glutaraldehyde solution, composed of 97.5 mL of cacodylate buffer and 2.5 mL of glutaraldehyde, was added to the dish to cover the cells. The glutaraldehyde solution was then removed using a pipette gun. The cells were maintained at room temperature for 2 h, prior to three washes using cacodylate buffer. After the last wash, the cells were covered with cacodylate buffer and stored at 4 °C prior to analysis [13, 16].

### Characterization of cells by immunoflow cytometry

Inverted light microscopy (≠Olympus, CKX41) was used to identify cultures containing confluent regions of adherent cells. Cells were detached with trypsin-EDTA (0.25 %), and then centrifuged at +4 °C three times, 5 min at 1200 rpm, washing with fresh medium each time. The pellets were resuspended in freshly prepared cell culture medium and transferred into falcon tubes. After cell counting (between 10^3^ and 10^6^), the cells were incubated with fluorescein isothiocyanate (FITC)- and phycoerythrin (PE)-conjugated monoclonal antibodies against cell surface markers (CD29, CD44, CD90, CD166, HLA-DR, CD10, CD11b, CD14, CD34, CD45, CD117), with appropriate controls, at 4 °C for 50 min, protected from the light. The cells were washed by the addition of PBS containing 0.1 % sodium azide, followed by centrifugation for 5 min at 1200 rpm. The supernatant was removed and the cells were resuspended in assay buffer and analyzed with a flow cytometer. Results were evaluated using the native software of the flow cytometer (Beckman Coulter Navious Software).

### Analysis

#### Inverted light microscopy

Chondrocyte and osteocytes were imaged using an inverted phase-contrast microscope at ×4, ×10, ×20, and ×40 magnifications, before and immediately after BA and MTT applications. Images were analyzed using the Olympus cell imaging software.

#### ESEM analysis

ESEM analysis was carried to assess the surface topography and composition of the cells. A device with a lifting system and the ability to transfer the electron beam in a high vacuum was used. This enabled us to obtain images of the extracellular matrix, as well as images of characteristic cellular structures. FEG ion pumps were used for the high vacuum. Images were recorded at a pressure of 100–220 Pa in ESEM vacuum mode, under magnifications of ×1000–×160,000, at resolution depths (HFW) of 41.4 and 414 μm, at an operating voltage of 5.00 kV, and at a WD of 8.8–8.9 mm.

#### MTT-ELISA vitality, toxicity, and MTT-proliferation analyses

Vitality tests were performed using a commercial MTT (3-[4,5-dimethyltiazol-2-yl]-2,5- diphenyltetrazolium bromide; Thiazolyl blue) kit, according to the manufacturer’s instructions. The working principle of the kit involves the cleavage of the tetrazolium ring by mitochondrial dehydrogenase enzymes, yielding blue formazan crystals, which are formed only in live cells [13–15, 17].

MTT assays were carried out on chondrocytes and osteocytes prior to addition of BAs. The same procedure was then repeated 24 and 48 h after the addition of BAs. The culture media containing BAs discarded from wells by a gun pipettor. Instead of removed supernatant, 12 mM/5-mg MTT tetrazolium solution, 1 mL sterile PBS, and DMEM were added, followed by SDS (prepared by adding 10-mL 0.01 M HCl to 1-g SDS), and then 100-μL MTT stock solution. Cultures were incubated at 37 °C for 2 h protected from the light. A 500-μL aliquot of the resultant reaction solution was removed for analysis. Dimethyl sulfoxide was added to these samples, which were incubated at 37 °C for an additional 10 min, prior to photometric analysis of a 540-nm wavelength absorbance. For assessment of proliferation, 500 μL of SDS-HCl solution was added to the remaining cells and incubated at 37 °C for 18 h. The reaction solution was subjected to photometric analysis at a 570-nm wavelength. The vitality of the control group was assumed to be 100 % prior to addition of BAs (0 h) to the culture medium.

Five different biological agents were applied to both chondrocyte and osteocyte cultures for 24 and 48 h. After this period of time, MTT was applied and the cells were incubated for an additional 12 h to monitor proliferation. Effect on the proliferation of the 24- and 48-h application of BA can be traced under an inverted microscope, obtaining images at the 36th and 60th hours. After the 60th hour, no live cells or proliferation were observed in the cultures, so all tests were terminated at this time point.

### Statistics

Cell numbers and proliferation rates were subjected to statistical analysis using the Minitable R15 software. To test for differences among the groups, variance analysis was used. Tukey’s test was used for significance differences among means (95.0 % confidence interval). The statistical significance level was set at *p* < 0.01.

## Results

### Light microscopy and immunoflow cytometry analysis of chondrocyte and osteocyte primary cell culture

**Fig. 2:**
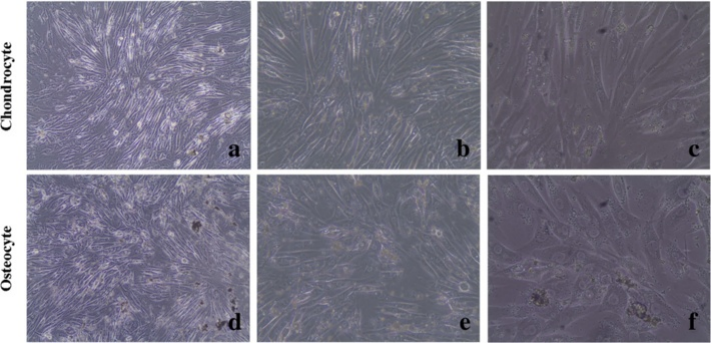
Inverted light microscopy images of chondrocytes and osteocytes cultures (**a**, **b**, and **c** are ×10, ×20, and ×40 magnification of approximately 97 % confluent chondrocytes, respectively; **d**, **e**, and **f** are ×10, ×20, and ×40 magnification of approximately 91 % confluent osteocytes, respectively)

Chondrocytes reached approximately 97 % confluence while osteocytes 91 % in primary culture conditions, indicating a healthy proliferative state (Fig. 2).

**Fig. 3:**
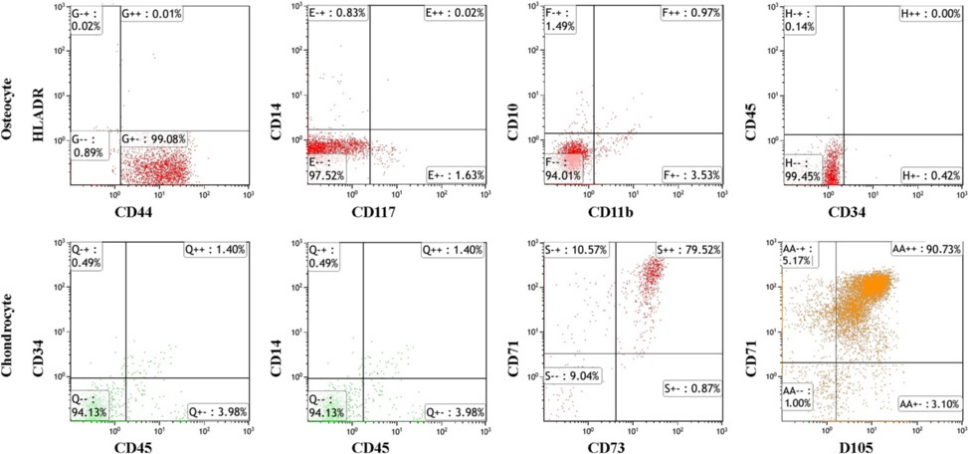
Immunoflow cytometric evaluation of cell surface antigens of osteocyte and chondrocyte

Immunoflow cytometry analyses demonstrate that osteocytes were negative for HLA-DR, CD10, CD11b, CD14, CD34, CD45, and CD117 (non-significant), but positive for CD44 (99.08 %). Chondrocytes did not express CD34, CD14, and CD45 (non-significant) surface markers, but did express CD71, CD73, and CD105 (90.73 %), all of which are typical markers for mesenchymal stem cells (Fig. 3).

**Fig. 4:**
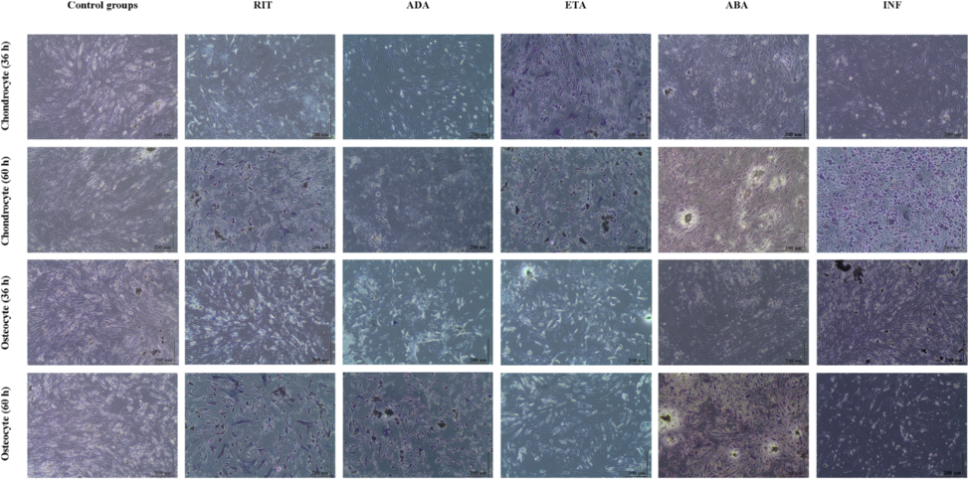
Proliferation of chondrocytes and osteocytes after 24 and 48 h BA application. Analysis of proliferation was performed in all test groups. After administration of BAs for 24 and 48 h, cultures were allowed for proliferation for an additional 12 h. After 24 h administration of BA, suppressed proliferation were showed by MTT assay performed at the 36th hour and complete blockage of proliferation after 48 h BA application were showed by MTT assay performed at the 60th hour, and absorbance values were obtained

The decrease in the number of both chondrocyte and osteocyte cells observed after 24 h BA application was compared with the control groups. In terms of the proliferation, 48 h application of any BAs could completely suppress proliferation at the dose levels studied (Fig. 4).

But, for some BAs, observed impact was more dramatic. The effect of BA was to suppress proliferation as well as changing the cell morphology and extracellular matrix formed in the culture conditions. Especially, for INF-applied chondrocytes, these effects were much more obvious, so that cell detachment and altered cell morphology were observed. However, an opposite situation was seen in osteocytes exposed to ETA, in that morphologically occurred in these cells.

### ESEM evaluation

**Fig. 5:**
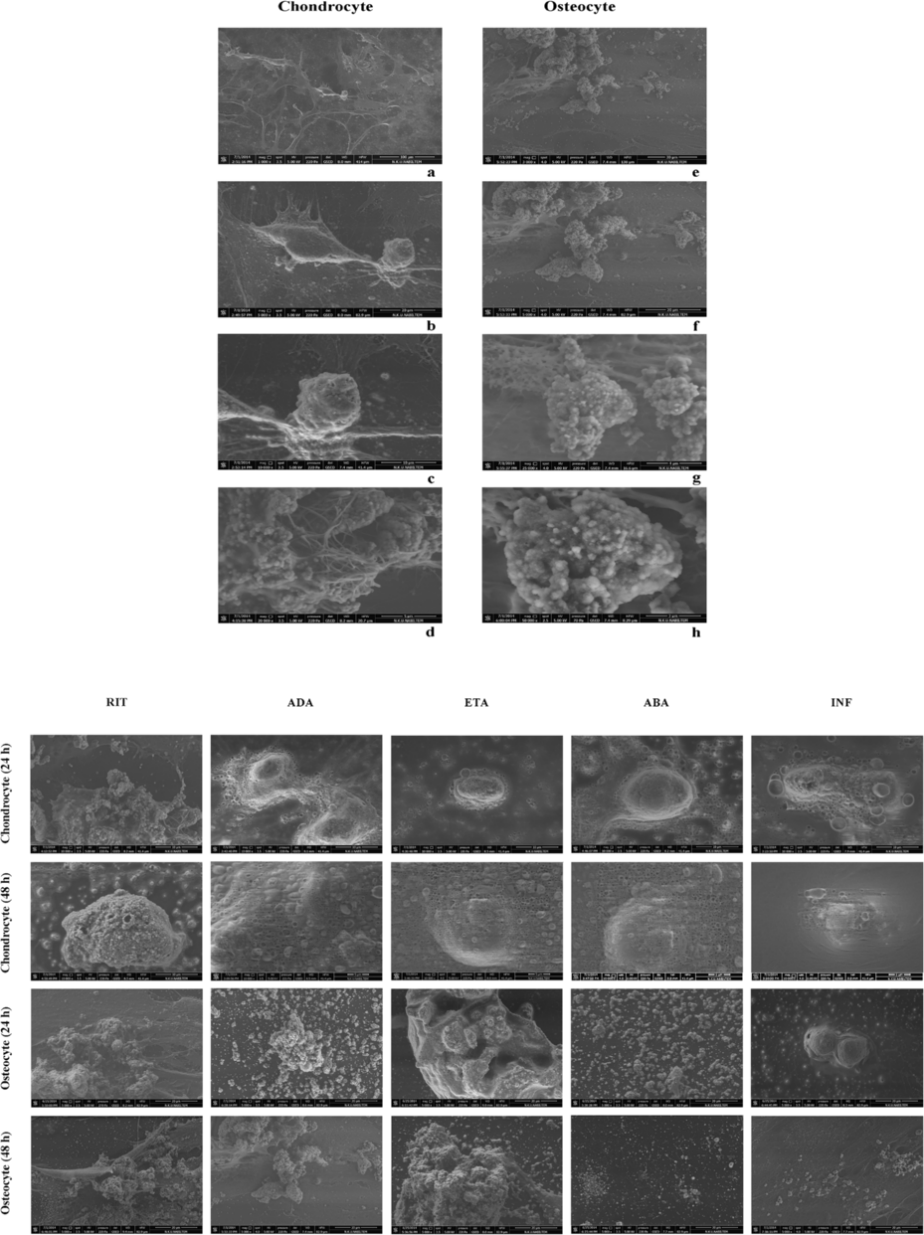
Morphological evaluation of cell surfaces. ESEM images of control group, chondrocyte, and osteocyte cultures (**a**, **b**, **c**, and **d** are ×1000, ×5000, ×10,000, and ×20,000 magnification of chondrocyte, respectively; **e**, **f**, **g**, and **h** are ×3000, ×5000, ×25,000, and ×50,000 magnification of osteocyte, respectively). ESEM images of chondrocytes after 24 and 48 h BAs application (magnification ×10,000) and osteocytes after 24 and 48 h following the administration of BAs (magnification ×5000)

In healthy chondrocytes and osteocytes showing osteoblastic activity, all natural surface characteristics are visible in the control group images. Viable cells were observed 24 h after application of RIT and ADA to chondrocyte cultures, and 24 h after application of ADA and ETA to osteocyte cultures. Deterioration of cell surface morphologies and loss of extracellular matrix were evident in chondrocyte cultures after 24 h ETA, ABA, and INF exposure, and osteocyte cultures after 24 h ABA, INF, and RIT exposure. Similar results were observed 48 h after the application. In all groups exposed to BAs, deterioration of the cell surface and loss of matrix were observed in cultures of both cell types (Fig. 5).

In all cases, dead cells were scattered throughout the culture, the number of dead or live cells was decreased, and cell morphologies were altered. In some images, cells appeared to be contracted and to have lost their specific morphology.

### Statistical evaluation of the effects of BAs on chondrocyte and osteocyte viability and proliferation

The MTT results supported our morphological observations. ESEM data were consistent with the results of the MTT-ELISA viability, toxicity, and MTT-proliferation data analyses, as well as the inverted light microscopy images. The images supported the MTT data corresponding to cartilage and bone cells before and 24 and 48 h after the application of BAs.

**Fig. 6:**
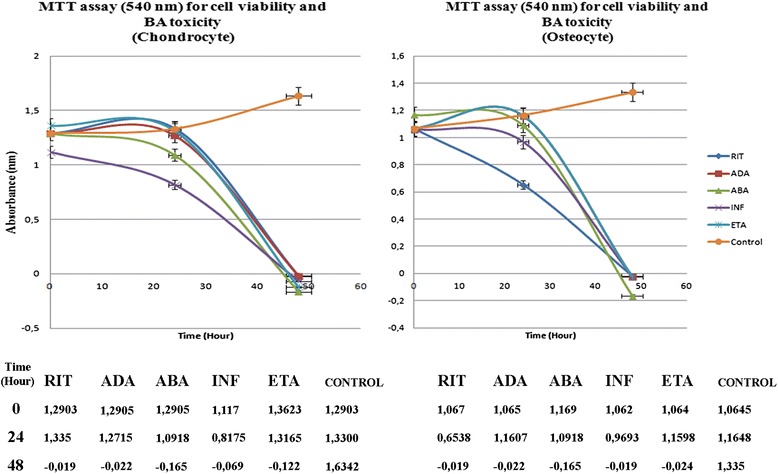
Cartilage and bone cell viability (MTT assay; 540-nm absorbance)

MTT analysis showed a decrease in cell viability in all groups after 24 h BA application, and no viable live cells remained after 48 h BA application. Obtained absorbance values are listed (Fig. 6).

**Fig. 7:**
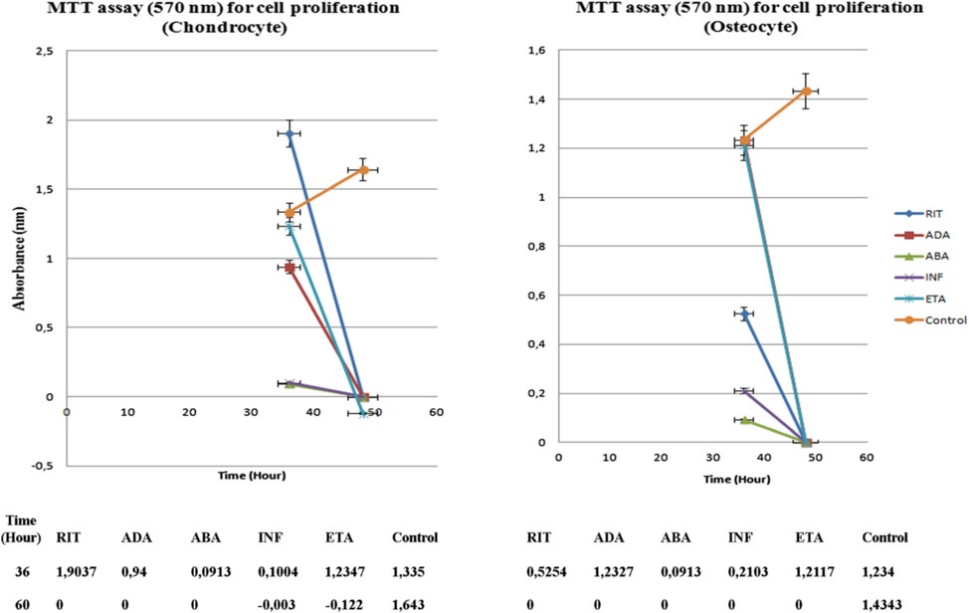
Cartilage and bone cell proliferation (MTT assay; 570-nm absorbance)

Analysis of proliferation clearly showed that in all test groups, 24 h application of BA suppressed proliferation (absorbance values obtained at the 36th hour) which was completely blocked after 48 h BA application (absorbance values obtained at the 60th hour) (Fig. 7).

At 24 h, in the RIT group, more viable chondrocyte cells were present, compared to the other groups (*p* < 0.01 [*p* = 0.0000]). RIT allowed chondrocyte proliferation up to 36 h (*p* < 0.01 [*p* = 0.0000]). In the same period of time, the viability rate of chondrocytes in the ADA group was similar to the RIT group (*p* < 0.01 [*p* = 0.0000]). In the ETA, ABA, and INF groups, the number of live chondrocytes was lower than that of the RIT, ADA, and control groups, suggesting that ETA, ABA, and INF inhibited chondrocyte proliferation (*p* < 0.01 [*p* = 0.0000]). Proliferation analysis performed 48 h after BA application showed no living cells or proliferation in all groups, other than the control group (*p* < 0.01 [*p* = 0.0000]).

In the osteocyte cultures, the number of live cells was the greatest in the ADA group, followed by the ETA group, 24 h after BA application, compared to the control and all other groups (*p* < 0.01 [*p* = 0.0000]). From the results of the proliferation test, which was made at the 24th hour, it was clear that proliferation occurred in ADA and ETA groups at 36th hour (*p* < 0.01 [*p* = 0.0000]).

The viability rate of osteocytes in the ABA, INF, and RIT groups was found to be very low compared to that in the control, ADA, and ETA groups, suggesting that these BAs inhibited osteocyte proliferation (*p* < 0.01 [*p* = 0.0000]).

The results of proliferation test at the 60th hour showed no proliferation in all BA groups, while proliferation in the control group was continued (*p* < 0.01 [*p* = 0.0000]).

## Discussion

The effectiveness of BAs in rheumatic diseases has been investigated since 2006, but still, there are only a few studies involving toxicity-related pre-clinical analyses which have been performed up to date [18]. The present study aimed to contribute to literature regarding the comparative toxic effects of BAs on osteochondral tissues.

With the development of therapeutics and the advancement of regenerative medicine, research into the molecular toxicity of medicines [19] and methods to restore damaged tissues [20] has increased dramatically. With the aim of protecting or restoring chondrocytes and osteocytes in the context of orthopedic surgery, studies have focused on tissue engineering, consistent with many other medical fields [13, 19, 21].

Recently, molecular toxicity analyses of drugs have increased in popularity [8–11, 22–25]. Orthopedic research also was influenced from this trend topic. The toxic effects of drugs on healthy chondrocytes and osteocytes have also been investigated, and researchers asserted the potential chondrotoxicity/osteotoxicity [26–29].

Although the BAs are normally brought to the market for oncology, after FDA’s approval in cases where the DMARDs are insufficient in rheumatic diseases, it began to be commonly prescribed. Especially in the active period of rheumatic diseases, these agents used to suppress inflammation, side effects, and/or adverse effects were encountered in literature [3, 8–11].

Even if a number of clinical studies especially in terms of side effects of BAs used in our study exist, we could not find *in vitro* experimental studies indicating effects on the articular cartilage and bone tissue in literature. In addition, to our best knowledge, any study comparing the effects of BAs on cell viability and proliferation of chondrocytes and osteocytes lacks in the literature. However, most reports up to date are based on experimental animal models, those comprising animal tissues [19]. Although studies based on animal models highlight differences between human and animal tissues, researchers may interpret data differently and draw differing conclusions [19].

Primary human chondrocytes and osteocytes obtained from osteochondral tissue explants have been used in experimental processes in this study. Studies using primary cultures are valuable since cell cultures contain all types of cells in the tissue, even the extracellular matrix components. The only limitations of such studies are the adjustment of the drug dose which will be applied to the culture medium. As it is well known, for systemically administered drugs, it is accepted that the concentration of drug is equal in all tissues and is metabolized at the same dose rate according to the virtual volume distribution rules [30, 31]. So, in primary chondrocytes and osteocytes explant cultures, we considered the dosages tested on other tissues and reached a peak concentration in the blood [32, 33].

In the present study, we aimed to observe the effects of three TNF inhibitors, ETA (soluble TNF receptor fusion protein (p75-IgG fusion protein), INF (chimeric anti-TNF monoclonal antibody), and ADA (recombinant human IgG1 monoclonal antibody), and two IL-1 antagonists, ABA (CTLA4-IgG1 fusion protein) and RIT (anti-CD20 monoclonal antibody; used in B-cell-inhibiting treatments) on human primary chondrocyte and osteocyte cultures. An additional aim was to evaluate the toxicity and effects of these BAs on the viability and proliferation of the cells in question.

Owing to difficulty in isolating osteocytes from the bone tissue, characterization of osteocytes and chondrocytes via immunoflow cytometry was performed before their use in the experiments. Immunophenotypic characterization of these cells is usually performed on expanded cells rather than primary cell cultures. Although no perfect marker has been defined for cells grown in culture, it is known that hematopoietic markers, such as CD34, CD14, and CD45, are not expressed. CD44, a receptor for ligands such as hyaluronan and osteopontin, is a marker of osteocytes, but there is no such a specific receptor for chondrocytes [17, 34]. For this reason, CD71, CD73, and CD105, which are typical markers with well-characterized expression, especially mesenchymal stem cells, were utilized [16].

Pharmacological alternatives of BAs used in this study, including bevacizumab, ranibizumab, aflibercept, and Ziv-aflibercept were studied on different tissues, and mild mitochondrial toxicity was reported [11]. Furthermore, the anti-TNF-α agent anakinra was tested, and the resulting specific side effects regardless of the dose were stated [23]. However, to our knowledge, there exists no in vitro study in the literature comparing the molecular effects of TNF inhibitors, IL-1 antagonists, and B-cell-depleting BAs on the viability, toxicity, and proliferation of chondrocytes and osteocytes.

The cases included in the study were those which confirm that the toxicity in osteocytes and chondrocytes was a result of BA therapy and not a patient-specific cellular response; we ensured that the patients from whom the tissue samples were provided were not exposed to methotrexate, fludarabine, cyclophosphamide, high-dose steroids, and/or an anti-pneumococcal vaccination, and that they did not have protein allergies or a history of RA.

In the present study, the data obtained at the 24 h showed that the number of live cells and the rate of chondrocyte proliferation were highest in the RIT group, and that ABA and INF were the most toxic to chondrocytes. However, at the 48 h, neither viable cells nor proliferation were observed in all groups, except the control (*p* < 0.01 [*p* = 0.0000]).

At the 24 h, the highest number of viable osteocytes was in the ADA group, followed by the ETA group. But, viability rates in the ABA, INF, and RIT groups were lower. Based on the proliferation analyses at the 48 h, similar to the chondrocyte cultures, there were no viable osteocytes and proliferation in all groups, except for the control. Osteotoxicity was highest in the RIT group, followed by the INF group (*p* < 0.01 [*p* = 0.0000]).

In all groups exposed to BAs for 24 h, when compared with the control group, MTT results showed that cell vitality decreases. For the same group of cells, when inverted light microscopy and ESEM images are evaluated together, a decrease in the number of viable cell concentration is observed compared to the control group. In addition, when images are examined, changed cell morphology, loss of the specific cell shape, and detachment from the extracellular matrix formed in the culture vessel are observed. The remaining live cells could not divide or proliferate after BA administration. The 48-h BA application completely inhibited vitality and proliferation of chondrocytes and osteocytes. Beyond morphological observations, statistical analysis of MTT data supports this data and was significant.

## Conclusion

RIT and ADA were found to be the least toxic biologics for cartilage, whereas ADA and ETA were the least toxic for bone cells (*p* < 0.01 [*p* = 0.0000]). Tested doses are determined based on the previous studies and systemic peak levels of patients. Our data indicate that chondrotoxicity and osteotoxicity of BAs should be taken into consideration on the treatment regimen during active inflammation periods.
